# Learning With Fewer Images via Image Clustering: Application to Intravascular OCT Image Segmentation

**DOI:** 10.1109/access.2021.3058890

**Published:** 2021-02-11

**Authors:** CHAITANYA KOLLURU, JUHWAN LEE, YAZAN GHARAIBEH, HIRAM G. BEZERRA, DAVID L. WILSON

**Affiliations:** 1 Department of Biomedical Engineering, Case Western Reserve University, Cleveland, OH 44106, USA; 2 Interventional Cardiology Center, Heart and Vascular Institute, The University of South Florida, Tampa, FL 33606, USA

**Keywords:** Annotation, deep learning, image clustering, optical coherence tomography, semantic segmentation

## Abstract

Deep learning based methods are routinely used to segment various structures of interest in varied medical imaging modalities. Acquiring annotations for a large number of images requires a skilled analyst, and the process is both time consuming and challenging. Our approach to reduce effort is to reduce the number of images needing detailed annotation. For intravascular optical coherence tomography (IVOCT) image pullbacks, we tested 10% to 100% of training images derived from two schemes: equally-spaced image subsampling and deep-learning- based image clustering. The first strategy involves selecting images at equally spaced intervals from the volume, accounting for the high spatial correlation between neighboring images. In clustering, we used an autoencoder to create a deep feature space representation, performed k-medoids clustering, and then used the cluster medians for training. For coronary calcifications, a baseline U-net model was trained on all images from volumes of interest (VOIs) and compared with fewer images from the sub-sampling strategies. For a given sampling ratio, the clustering based method performed better or similar as compared to the equally spaced sampling approach (e.g., with 10% of images, mean F1 score for calcific class increased from 0.52 to 0.63, with equal spacing and with clustering, respectively). Additionally, for a fixed number of training images, sampling images from more VOIs performed better than otherwise. In conclusion, we recommend the clustering based approach to annotate a small fraction of images, creating a baseline model, which potentially can be improved further by annotating images selected using methods described in active learning research.

## I. INTRODUCTION

For deep learning applications in medical imaging, there is a need to conserve the numbers of images required and the numbers of images requiring detailed annotation. In medical imaging, deep neural networks have been used in all areas of image processing/analysis, including image segmentation, enhancement, restoration, classification, and reconstruction. Here, we focus on the image segmentation problem, but results could be applicable to other problems as well. Unlike applications using natural images where hundreds of thousands or even millions of images can be used, most often many fewer images are available in medical imaging, suggesting a need to learn as much as possible from available images. In addition, for semantic segmentation, images must be annotated, oftentimes a time consuming and difficult task, requiring expensive experts. For example, here we examine segmentation of coronary calcifications in intravascular optical coherence tomography (IVOCT) images. IVOCT is an imaging modality that allows for assessment of coronary plaque burden during interventional treatments of coronary artery disease. A typical IVOCT pullback volume generates nearly 300–500 image frames. Thus, very significant manual effort is required to deeply annotate even a single pullback. In addition, there is a very significant variability between lesions, much more than that found, for example, in organ segmentation, suggesting the need to train on a very large number of lesions.

To reduce annotation effort, two methodologies have been proposed in literature [1]. The first method aims to work in cases with scarce annotations, where only a limited set of images are densely annotated. Examples of this technique include data augmentation [2], shape regularization [3], domain adaptation [4], [5], supervised pretraining [6] and active learning [7]. The second method involves creating models that work in cases of weak annotations, where only sparse or image- level labels exist. Few examples of this method include utilizing class activation maps from a classification task to train a segmentation network [8], [9], mask completion methods such as Grabcut algorithm [10] among others. Since semi-supervised generation of pixel-wise ground truth labels from weak annotations is prone to errors, the former method of using scarce annotations is usually preferred. There are important constraints in medical imaging - segmentations must be accurate and there can be a limited number of images as compared to non-medical applications.

Active learning presents a method to select candidate images for annotation when only scarce labels can be provided. Such methods work in an iterative manner to select unlabeled images for annotation at each stage to improve model performance. However, these techniques require an initial trained model, thus necessitating some expert-annotated labels beforehand.

Further, in a scenario where annotations are expensive, the developer of a segmentation model faces a few intriguing questions. Firstly, if an expert has identified volumes of interest (VOI) within an image volume for learning, is it necessary to label all images in the VOI to train a model or is a smaller subset sufficient? Secondly, if only a small percentage of the images can be annotated, which images should be selected for expert annotation? Specifically, when applying 2D segmentation networks in a slice-by-slice manner to 3D datasets, is there a method to sample images from a VOI that performs better than sampling at equally spaced intervals across the volume? Thirdly, for a fixed number of training images n, does an image sampling strategy that subsamples from all VOIs perform better than selecting the first n images, effectively considering a smaller number of VOIs?

In this work, we present a deep-feature based image clustering technique to select images from an expert-identified VOI for detailed annotation. We conducted experiments to segment calcification lesions in a dataset of clinical IVOCT scans. A report by Zheng *et al.* [11] applied such a sampling strategy to sparsely annotate images in a volume and train a 3D network that works with sparse annotations. However, since the number of slices in a VOI can vary significantly depending on the lesion size (range: 10–200 slices in our dataset), applying a 2D segmentation network in a slice-by-slice manner is more intuitive. Clustering using deep-features from an autoencoder trained on images within a VOI eliminates the need for an initial set of labels as in active learning based methods.

To test our hypotheses and answer the questions detailed above, we created seven different segmentation models, each trained on a different training set. Briefly, a baseline model using all images in the VOI for training was constructed first. Next, we trained three models, each considering images sampled from the VOI at equal spacing but with different sampling ratios (one-half, one-third and one-tenth). Finally, we trained three models using images sampled from the VOI with a clustering based approach at the same sampling ratios described earlier. Model performance was evaluated on a held- out test set. We folded this process over the entire dataset to reduce selection bias, ensuring that each image in our dataset becomes a part of the held-out test set exactly once.

## II. IMAGE ANALYSIS METHODS

**FIGURE 1.**
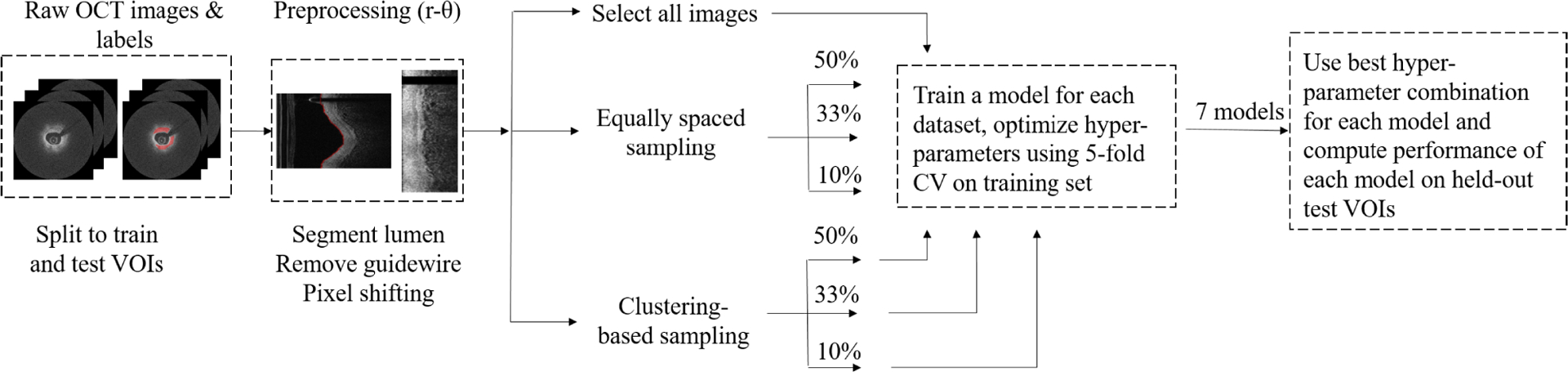
Schematic illustration of overall approach. After preprocessing images in the r-*θ* domain, our pipeline consists of three main branches. In the first branch, a segmentation model is trained using all images from each VOI. The second branch creates three segmentation models, each trained on a subset of images from each VOI, where images are sampled at equal spacing (in z) across the volume. Finally, the third branch creates models trained on a subset of images from each VOI as well, but an image clustering approach is followed for image selection. Percentages on the arrows indicate percentage of images selected from each VOI.

Image processing and deep learning based 2D segmentation networks are used to classify pixels in IVOCT images as calcific or other. We will use the following naming convention throughout the rest of the paper: pixels belonging to an expert- annotated calcification will be referred to as calcific and all remaining pixels will be referred to as other. The algorithm can be broken down into three main steps (Fig. 1): (1) image preprocessing, involving lumen segmentation, guidewire identification, tissue alignment by pixel shifting and speckle reduction; (2) optional selection of images from each VOI in the training dataset, where images were sampled either at equal spacing along the z-stack or by an automatic image clustering method; (3) training and testing a deep neural network for the task of image segmentation.

### A. PREPROCESSING

Raw IVOCT images in the polar (r, *θ*) domain are preprocessed to aid in the task of image segmentation. Preprocessing steps are described in detail in our previous work [12], and are summarized here. Briefly, the lumen boundary is found using a deep learning based technique proposed by our group [13]. This is followed by detection of A-lines within the guidewire shadow and setting pixels within these A-lines to zero since they do not contain any information accounting for the strong light reflection at the guidewire. Next, A-lines are pixel shifted in the radial direction to align tissues, correcting images with either non-circular lumen or eccentric catheter position. Images are cropped in the radial direction to consider only the first 200 pixels (~1 mm) from the lumen giving a fixed image size. Subsequently, a log transform is applied followed by Gaussian filtering with a kernel of size (7, 7) and standard deviation 1 to reduce speckle noise.

### B. IMAGE SUBSET SELECTION

We consider two methods to subsample images for network training. These sampling methods are performed only on the VOIs within the training dataset. The first method involved selecting images at equal z-spacing in the volume. For example, if the sampling ratio is set to be one-third, every third image in the ordered z-stack is considered. We refer to such a sampling strategy as equal spacing in the remainder of this paper. The second method involves an image-clustering based approach, is referred to as clustering, and is detailed below.

Deep-feature-based image clustering provides a method to select images that vary in their deep feature space representation. Images within each VOI are used as training samples for an auto-encoder with the same network architecture as the segmentation network. Processing has a number of steps. Firstly, the auto-encoder is trained for a fixed number of epochs using all images in the VOI. Secondly, a forward pass is performed using the trained auto-encoder and feature maps from the bottleneck layer are collected, thereby resulting in a feature vector representation for each input image. Thirdly, Principal Components Analysis (PCA) is performed to further reduce the dimensionality of the bottleneck features while ensuring that the new feature space explains most of the variance present in the original feature space. Fourth, a k-medoids clustering algorithm with cluster count set to the number of images required to be subsampled from the VOI is applied in the reduced feature space. Images corresponding to cluster medoids are selected to train the segmentation network.

### C. DEEP NEURAL NETWORK FOR IMAGE SEGMENTATION

We utilize a common deep neural network architecture, U- net [14] for this work. Briefly, a VGG16 [15] encoder architecture is used in the contracting path of the U-net with the corresponding inverted architecture and skip connections in the expanding part. Encoder weights are pre-trained based on the ImageNet database [16] and fine-tuned when training on our dataset. Since we have a binary classification problem to distinguish pixels between calcific and other, a sigmoid activation function is used to generate probability values from 0 to 1 for each pixel.


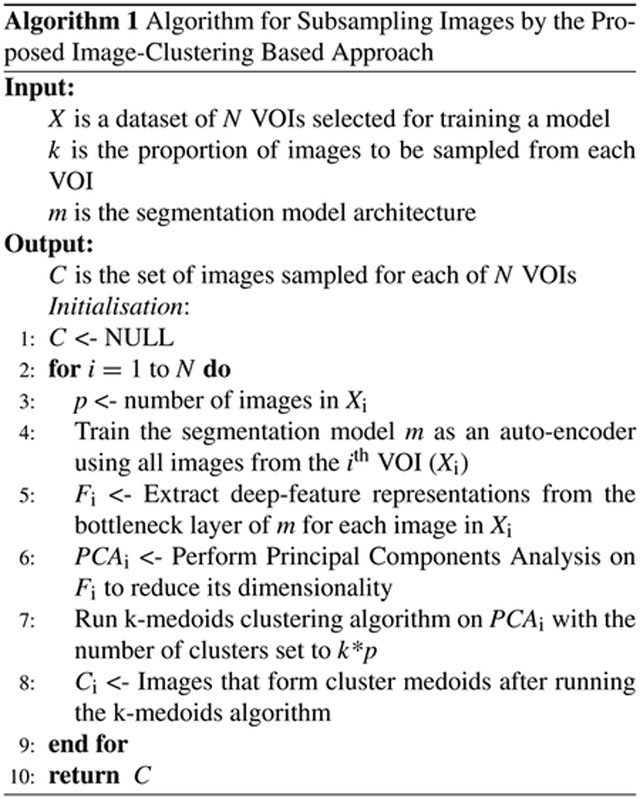


## III. EXPERIMENTAL METHODS

### A. LABELED IMAGE DATA

A dataset of 41 IVOCT pullbacks were used. All pullbacks were imaged prior to any stent intervention. Details about the IVOCT imaging system such as wavelength range, frame rate, pullback speed and axial resolution are described in detail in our previous work [12]. A total of 3,741 image frames from 60 VOIs were used in this study. Pixel-wise calcium segmentations were created by two expert readers trained in the Cardiovascular Imaging Core Lab of the Harrington Heart and Vascular Institute at University Hospitals (Cleveland, Ohio). Calcifications were identified using well-defined IVOCT attributes from the literature [17], viz., a signal poor region with sharply delineated front and/or back borders.

### B. IMAGE CLUSTERING

**FIGURE 2.**
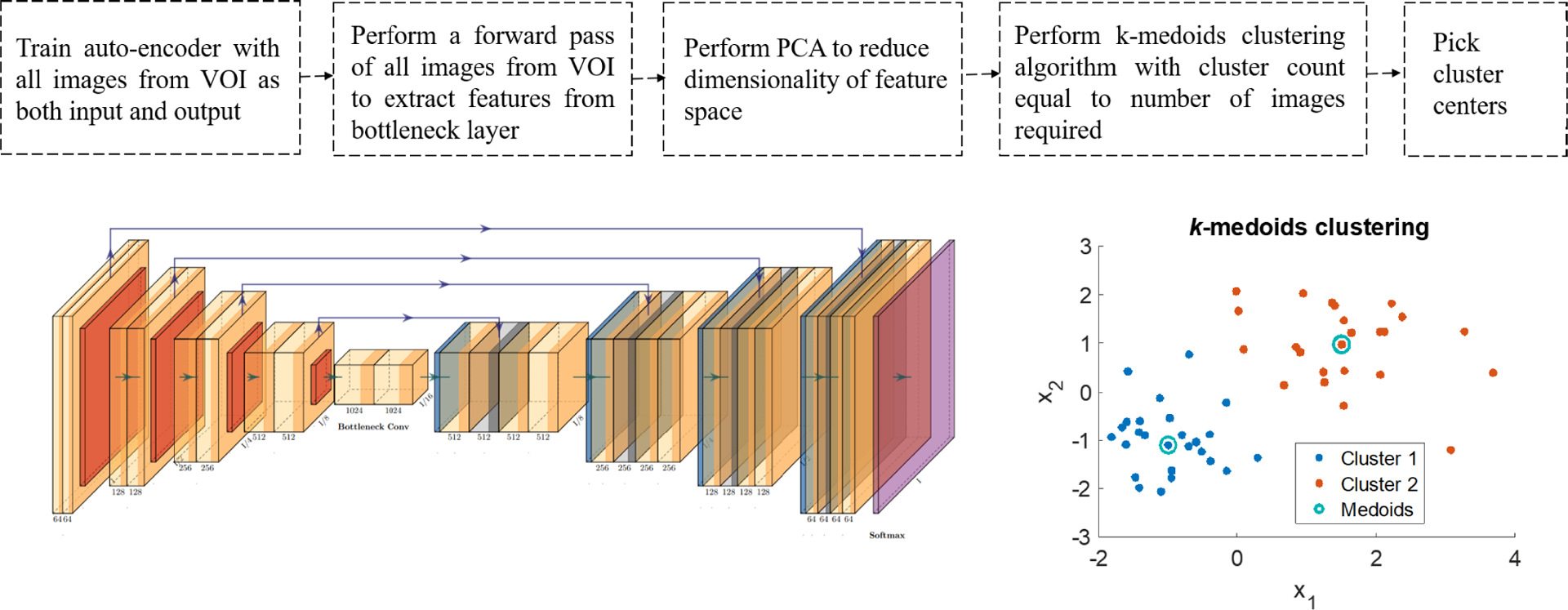
Overview of image clustering approach. Deep-learning based features are extracted and used for k-medoids clustering. Cluster centers (medoids) are selected and used for training. Colors in the auto-encoder indicate layer types: convolutional layers are marked yellow, max pooling layers are red, and upsampling layers are blue.

Features generated from a trained auto-encoder were used to perform unsupervised clustering. Briefly, all OCT images in a VOI were used both as input images and as labels for training an auto-encoder with a U-net architecture. The auto-encoder was trained for 100 epochs with learning rate of 0.001 and batch size of 64 for each VOI. This set of hyper-parameters were chosen based on visual inspection of reconstruction results. Post training, images in the VOI were fed through the network and feature maps from the bottleneck layers were extracted to create deep feature space representations. PCA was performed on the extracted feature vectors such that the resulting lower dimensional feature space could explain at least 95% of the variance in the original dataset. Finally, a k-medoids algorithm with the cluster number set to the number of images required to be selected from the VOI was performed on the lower dimension feature space and images forming the cluster centers (medoids) were selected. This is detailed in Fig. 2.

### C. NETWORK TRAINING AND TESTING

**FIGURE 3.**
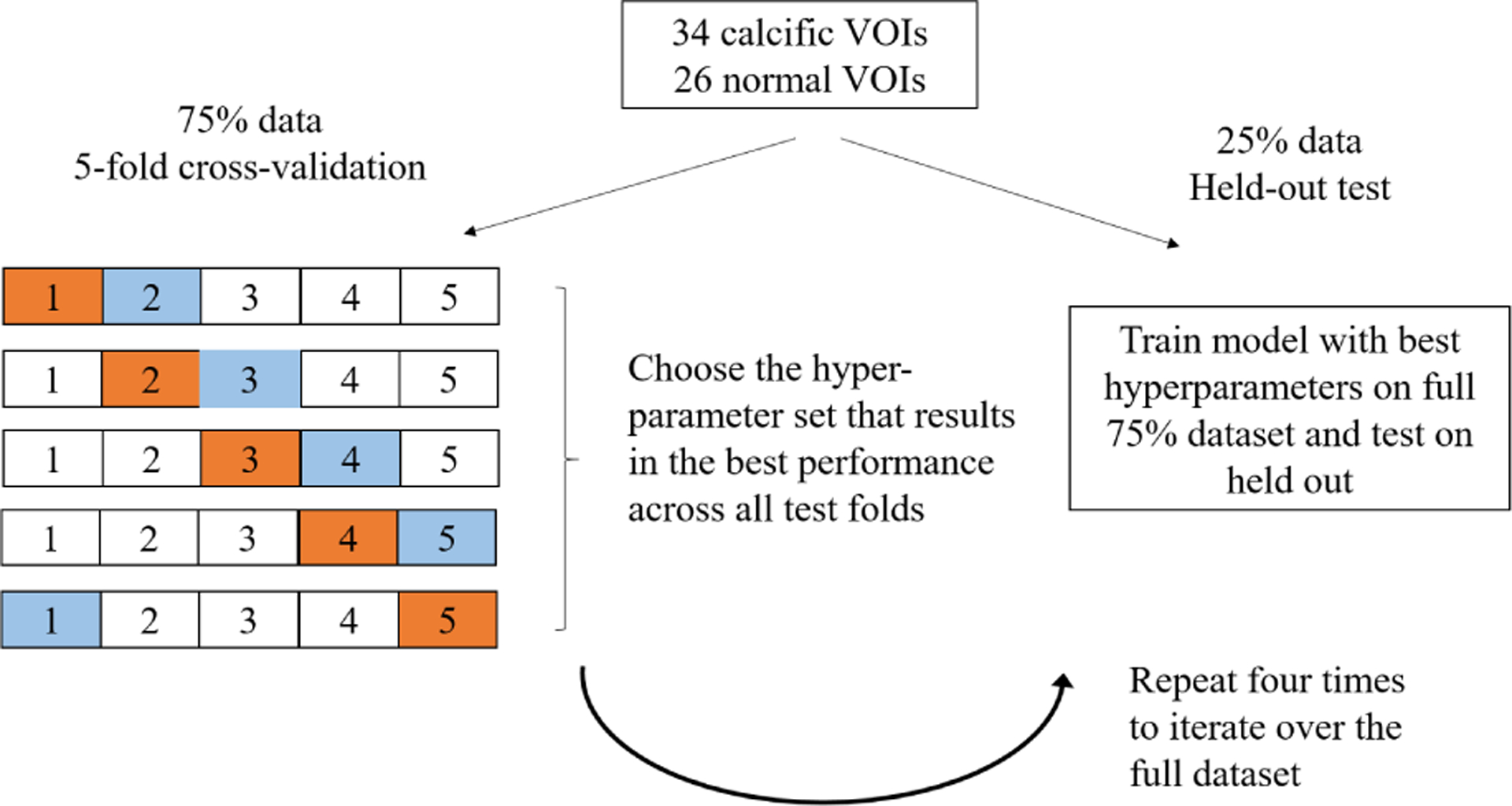
Training, validation, and test strategy for each segmentation model. The process is repeated for each image selection method and results are compared on the held-out test set. In the schematic shown above, orange indicates the test-fold and blue indicates the validation fold in each iteration.

We followed a 5-fold cross validation approach to optimize network hyper-parameters. Out of the 60 VOIs considered in the study, 26 VOIs were annotated normal (no clinically significant lesion) and 34 VOIs were annotated calcification by the expert analysts. Roughly 25% of VOIs were selected and placed in the held-out test set and the remaining VOIs were used for training. Training VOIs were subsequently split into one of five folds in a stratified manner. In each iteration of the 5-fold CV approach, three folds were considered for training, one for validation and one for testing. The validation set was used to stop network from overfitting on the training dataset, wherein network training was stopped when the loss on the validation set did not improve for 5 consecutive epochs. We used the Adam optimizer [18] and weighted binary cross-entropy as our loss function. Weights used in the loss function were inversely proportional to class prevalence and summed to one. Network hyper-parameters such as batch size and learning rate were optimized using a grid search approach, and best hyper- parameters were selected based on the mean performance across the test folds. The network was subsequently trained using the best hyper-parameter set to create a model for the full training dataset which was tested on the held-out test VOIs. The number of epochs needed to train the final model on the full training dataset was set to the average number of epochs run in different iterations of the 5-fold CV. F1 scores and area under the precision recall graph (average precision, AP) are recorded. We use precision recall curves instead of ROC curves since our dataset is highly imbalanced, consisting of roughly 10% calcific pixels and 90% other pixels. Since our selection of VOIs into the held-out-test set could result in biased estimates, we repeated the procedure described above four times, selecting a different set of VOIs into the held-out test set each time. In this manner, we ensured that each VOI in the full dataset becomes a part of the held-out test set exactly once (see Fig. 3).

In all, seven different trained models were created with different numbers of images used for training. Firstly, we trained with all the images in each VOI, giving a segmentation model called all. This replicates previous semantic segmentation studies from our group and others [12], [13], [19]–[22], and thus acts as a baseline for comparison. Next, three other models were trained with equally spaced image sampling in each VOI. The spacing gave one-half, one-third, and one-tenth the total number of images. These models allowed us to test the hypothesis that nearby, correlated images were sufficiently similar to add little value to training. The last three models were trained on images selected using the automatic clustering approach. For each VOI, the number of clusters used were one- half, one-third or one-tenth of the original number of images in the VOI. This final set of models allowed us to test the hypotheses that a significant difference in model performance exists between the image clustering based selection method and the equal spaced selection methods. Since this process was repeated four times on different held-out test sets, each image subsampling approach resulted in four trained models, and thereby four values for each performance metric.

We conducted an additional experiment to examine the effect of using a lower number of VOIs to select the same number of images. In this case, we considered one-tenth-clustered as our baseline model for comparison in each of the four iterations. We randomly shuffled the order of VOIs and collected all images from the VOIs, one after another in a serial manner until the desired number of images (one-tenth of total number of images in this case) was reached. Ten different models were created in each iteration, each training on images selected after a new shuffling operation on the order of VOIs. Results of the ten different models were compared to the respective one-tenth- clustered baseline model in each iteration based on the AP metric.

## IV. RESULTS

Image preprocessing methods based on knowledge of vessel morphology and OCT image acquisition allowed to us to segment and align tissue regions surrounding the vessel lumen. Such preprocessing allows the network to completely remove the clear luminal region from consideration when segmenting calcification regions. The auto-encoder used in the deep-feature extraction step was checked for reconstruction accuracy for each VOI. We found excellent reconstruction accuracy, with mean squared error between input and output images to be less than 0.03 in most cases (Supplementary Material S1). Best hyperparameter set selected from grid search for each of the seven models is provided in Supplementary Material S2.

We compared trained models based on the F1 score for calcific and other class. Mean and standard error for the F1 scores across the four iterations are reported in Table 1. Additional metrics are provided in Supplementary Material S3. We found that with one-half sampling, both the equally spaced and clustering approaches perform similar to using all images. However, as sampling ratio was reduced to one-tenth, we found that the clustering approach performs better than the equally spaced strategy, with higher mean F1 scores for both classes. Additionally, we found that the performance metrics of models trained using the equally spaced strategy with one-tenth sampling ratio had a large standard error.

**FIGURE 4.**
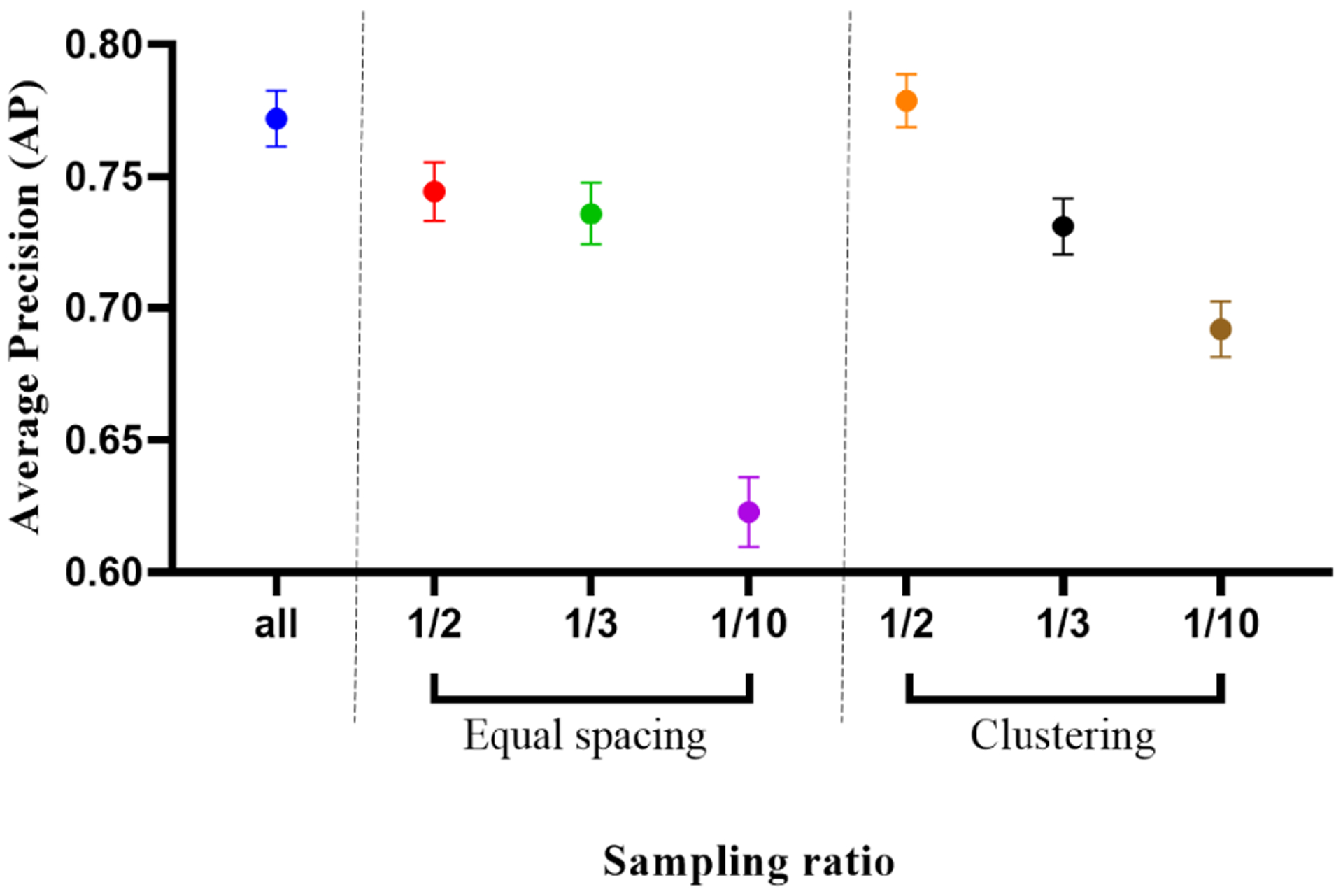
Mean and 95% CI of average precision (AP) score, considering each calcification containing frame in the held-out test set as an independent sample.

We also compared average precision (AP) metric for the seven different models. In this case, we grouped results from the four iterations together, given that no overlap exists between the four held-out test sets. We removed images from normal VOIs into this calculation since these images do not contain any pixels belonging to the positive (calcific) class, which makes the precision recall curve undefined. Consequently, considering each calcification image as an independent sample, we plot mean and 95% confidence interval estimates of the AP metric for each of the seven models described earlier (see Fig. 4). When we compared using clustering with one-half sampling to using all images, we found a statistically significant difference (p<0.05, paired t-test) with a higher mean. Models trained on the clustering based approach were found to have similar or better performance as compared to the equally spaced sampling approach for all sampling ratios (one-half: p<0.0001, one-third: no significant difference, one-tenth: p<0.0001, n = 1987, paired t-tests).

**FIGURE 5.**
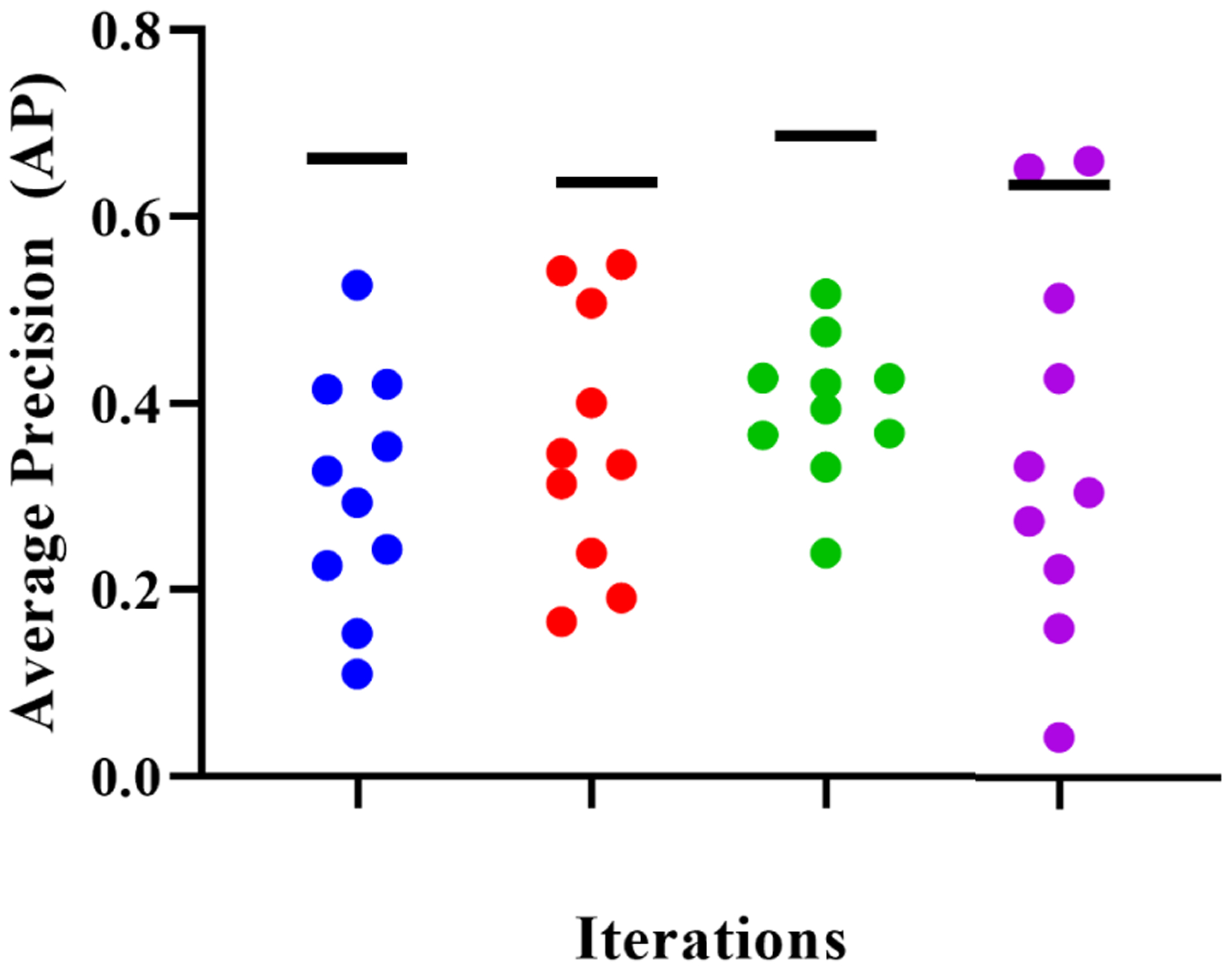
Performance comparisons between one-tenth clustered models (black horizontal lines) and models trained on the same number of images (one-tenth), but from a smaller number of VOIs (colored circles) in each of the four iterations. Colors in the graph distinguish models based on the iteration they were created in.

We compared AP scores from the ten models created from training on all images in a smaller set of VOIs to the AP score of the one-tenth clustered model in each iteration. This allowed us to test the hypothesis that when a fixed amount of images can be labeled (say one-tenth), selecting a subset of images from a larger number of VOIs performs better than selecting all images from a smaller set of VOIs. A one-sample Wilcoxon test showed that using images from a lower number of VOIs resulted in a lower performance in all iterations (p<0.005, see Fig. 5).

Finally, we visualized segmentations from one of our models with the ground truth in a few ways. We performed a Bland Altman analysis for various clinical calcification attributes (calcification angle, depth from lumen boundary and calcification thickness) between the best segmentation model in a particular iteration (one-third clustering) and the ground truth. Mean difference between predictions and ground truth was close to zero in all cases, indicating low bias (Supplementary Material S4). We also converted the images back to the XY (anatomical) representation and create fly through videos across a held out stack (Supplementary Material S5). Raw probability maps from all models for a held-out test VOI are shown in Supplementary Material S6.

## V. DISCUSSION

Our study examines methods for reducing the number of images needing annotation and compares the performance of a segmentation model when trained with either all images in the training VOI, or with a smaller fraction of images. In the latter case, we use two different sampling strategies to select images from a VOI. Firstly, we used a simple approach of selecting images equally spaced out in the VOI since it is known that slices form a contiguous volume within an OCT pullback, and thus slices close to each other are highly spatially correlated. Secondly, we implement a clustering approach using deep features to select images with distinct representations in the feature space.

Our results suggest ways to limit the significant effort required to annotate images for applications having great image variability. We found that the performance of the baseline model using all images from the training VOIs (all) is comparable to previously published results [13], [20], ensuring validity. For example, our baseline model (“all”) achieves a mean F1 score of 0.74 for the calcific class which is comparable to 0.73 reported in [13] and 0.72 reported in [20]. We found that with the clustering or equally spaced sampling approach within selected VOIs, one could reduce the number of annotations to one-half without significant changes in performance as compared to using all images. Additionally, we achieve similar results when an alternate network architecture for semantic segmentation was used (Feature Pyramid Network, proposed by Lin *et al.* [23]). These results are shown in Supplementary Material S7.

As we can obtain datasets with a large number of images, we are interested in getting the best models with far fewer image annotations. Let’s assume 200 pullbacks, and identification of 250 VOIs with and without calcifications giving about 10,000 images with calcification and 10,000 without calcification. Manual annotation of all images with calcification would require at least 160 hours. If we want to limit annotation time to say 20 hours of annotation, then we need to reduce the number of images annotated by a factor of 1/10. One method would be to simply analyze the first 2000 images. However, this greatly degrades performance as described earlier. Clustering would be the preferred method probably giving performance similar to using all images with these increased numbers of images. (That is, although there is degradation in performance with 10% of the about 4000 images used in these experiments, the relative amount of degradation will decrease as more learning samples are included, due to saturation of the learning curve.) Equal sampling would give much degraded performance. Continuing with the problem of getting the best performance from a limited number of annotations, one could create a reasonable baseline model from the limited annotations and identify additional images to annotate using methods reported for active learning [24]–[26].

Computation time for selecting images with the clustering approach was reasonable. Although clustering does add an additional computational cost in the system, training of the auto-encoder is complete in less than two minutes for a VOI of nearly 100 images. Upon further numerical experimentation, we found that training the auto-encoder for more epochs marginally improved reconstruction accuracy but increased computation time. K-medoids clustering and selection of median cluster centers is on the order of a few seconds, thus resulting in the clustering based subset selection approach to execute in well under two minutes for a VOI. For comparison, hyper-parameter optimization along with network training and testing took about 4–5 hours for each model. As noted earlier, feature extraction from the auto-encoder is a one-time step, and image features can be saved if clustering had to be repeated.

Our method can be improved in a few ways. Firstly, our clustering algorithm requires the user to specify the number of clusters to be created. In this work, we have set the number of clusters as a fixed percentage of the number of images in a VOI, which could be set based on expert availability. However, it is possible that the optimal number of clusters may vary from one VOI to another. To address this issue, it is possible to extend our work to identify the optimal number of clusters using methods described in literature. For example, techniques such as the average silhouette method [27], the gap statistic [28] allow users to identify the optimal number of clusters within a dataset. Secondly, our work does not include augmentation techniques so as to isolate the effect of data selection. We believe that it is possible to get performance gains across all models when training images are sent through an augmentation pipeline. In fact, if we extend our clustering approach to select two or three consecutive images at a time instead of one, a sliding window approach in the polar domain can be used to create many augmented images due to the nature of IVOCT image acquisition [13].

## Supplementary Material

access-3058890-mm

## Figures and Tables

**TABLE 1.** Performance metrics of all models. Metrics were calculated over all images in each of the four held-out test sets and mean and standard error across the four iterations are reported.

	F1-calcific	F1-other	Average Precision (AP)
all	0.74 +/− 0.01	0.97 +/− 0.01	0.77 +/− 0.01
one-half equal spacing	0.72 +/− 0.01	0.97 +/− 0.01	0.74 +/− 0.01
one-third equal spacing	0.67 +/− 0.04	0.97 +/− 0.01	0.71 +/− 0.04
one-tenth equal spacing	0.52 +/− 0.14	0.83 +/− 0.13	0.50 +/− 0.16
one-half clustered	0.73 +/− 0.01	0.97 +/− 0.01	0.76 +/− 0.03
one-third clustered	0.69 +/− 0.04	0.97 +/− 0.01	0.71 +/− 0.05
one-tenth clustered	0.63 +/− 0.01	0.96 +/− 0.01	0.66 +/− 0.01
