# Supplementary material for: Learning With Fewer Images via Image Clustering: Application to Intravascular OCT Image Segmentation
Source: IEEE Access. Author manuscript; Available in PMC 2021 Apr 6. (PMC8023588; doi:10.1109/access.2021.3058890)
Supplement: access-3058890-mm [file NIHMS1681423-supplement-access-3058890-mm.zip › access-3058890-mm/README.pdf]

DESCRIPTION: This file contains 6 MP4 files with 1 doc file with descriptions for each videos.

SIZE: 5.83 MB

PLAYER INFORMATION: MP4 files and doc file

PACKING LIST: access-3058890-mm.zip

CONTACT INFORMATION:

David L. Wilson, PhD
Robert Herbold Professor of Biomedical Engineering & Radiology
Case Western Reserve University
Cleveland, OH, USA 44106
Email：david.wilson@case.edu
