# Supplementary material for: Learning With Fewer Images via Image Clustering: Application to Intravascular OCT Image Segmentation
Source: IEEE Access. Author manuscript; Available in PMC 2021 Apr 6. (PMC8023588; doi:10.1109/access.2021.3058890)

Learning with fewer images via clustering: application to intravascular OCT image segmentation

Chaitanya Kolluru^1^, Juhwan Lee^1^, Yazan Gharaibeh^1^, Hiram G. Bezerra^2^, and David L. Wilson^1^

^1^Department of Biomedical Engineering, Case Western Reserve University, Cleveland, OH 44106 USA

^2^Interventional Cardiology Center, Heart and Vascular Institute, University of South Florida, Tampa, FL 33606 USA

Supplementary Material S1 (movie, mp4)

DESCRIPTION: Reconstruction results from the trained autoencoder on an example volume of interest (VOI). Preprocessed OCT volume provided as autoencoder input is shown on the left by single stepping through the image volume in the video. Reconstruction result (autoencoder output) is displayed on the right.

Supplementary Material S2

DESCRIPTION: Best hyper-parameter set (ordered as learning rate, batch size and epochs respectively) for all segmentation models created in this work.


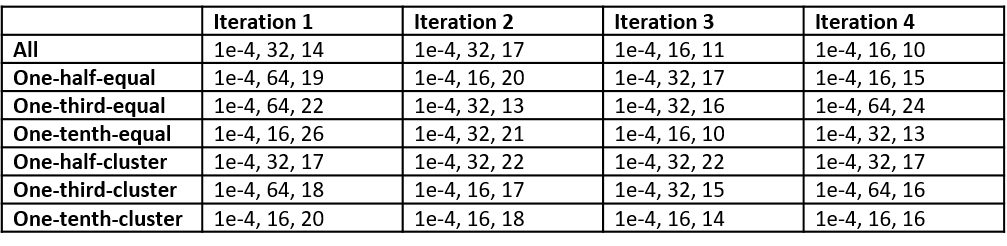


Supplementary Material S3

DESCRIPTION: Additional performance metrics of all models created in this work. Metrics were calculated over all images in each of the four held-out test sets. Metrics such as F1-scores and Average Precision are reported in Table 1 in the main text of the manuscript.


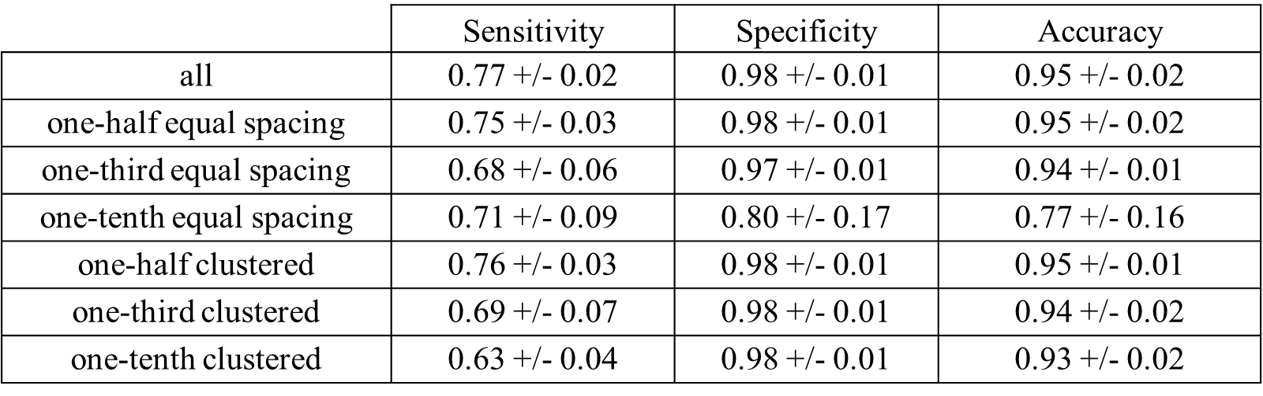


Supplementary Material S4

DESCRIPTION: Bland Altman analysis for various clinical calcification attributes such as calcification angle, depth from lumen boundary and calcification thickness between the best performing segmentation model in a particular iteration (one-third clustering) and the ground truth.


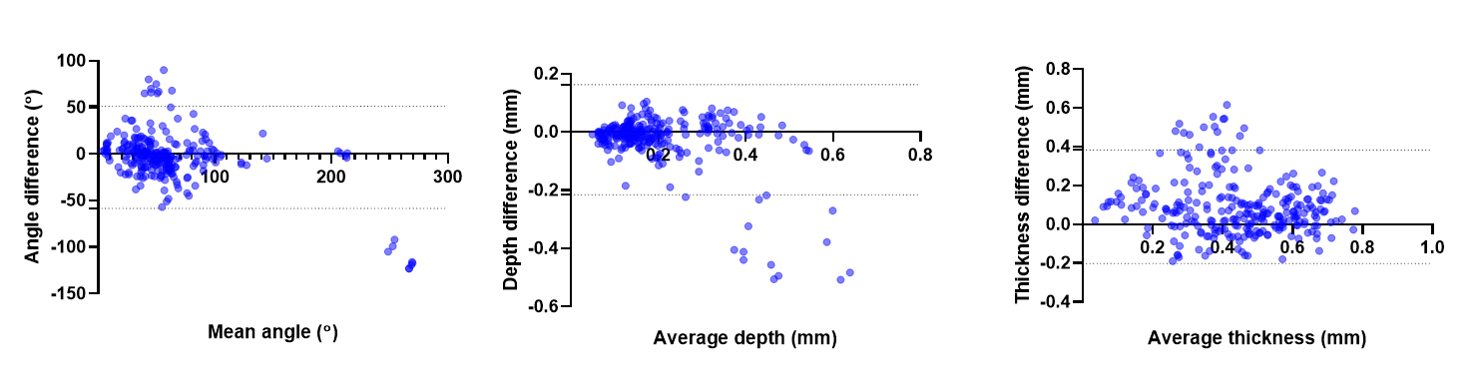


Supplementary Material S5 (movie, mp4)

DESCRIPTION: Results on a held-out test VOI displayed in the anatomical representation. The following image stacks are displayed: original IVOCT volume (left), IVOCT volume overlaid with ground truth labels in white (middle) and IVOCT volume overlaid with the best performing segmentation model in a particular iteration (one-third-clustering) in white (right).

Supplementary Material S6 (movie, mp4)

DESCRIPTION: Probability maps resulting from the segmentation network on a held out test VOI for each segmentation model. Segmentation performance is improved in the one-tenth clustering model when compared to the one-tenth equal spacing model.

Supplementary Material S7

DESCRIPTION: Performance metrics of all models when trained with an alternate network architecture (Feature Pyramid Network, Lin et al. 2017). Metrics were calculated over all images in each of the four held-out test sets and mean and standard error across the four iterations are reported. Similar conclusions can be made with this architecture, training with one-half of the images yields results comparable to using all images. At sampling ratios of one-third and one-tenth, the clustering based methods perform better than the equally spaced sampling counterpart.


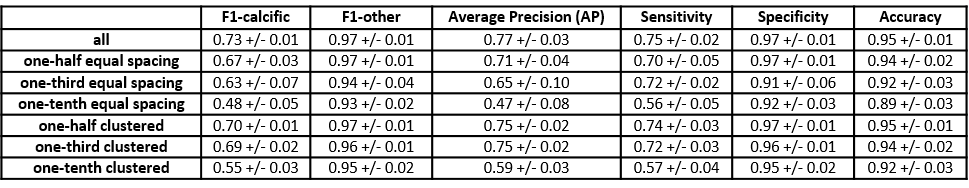

Supplement: access-3058890-mm [file NIHMS1681423-supplement-access-3058890-mm.zip › access-3058890-mm/Supplementary Information ACCESS3058890.docx]
