# Supplementary figures and images for: Learning With Fewer Images via Image Clustering: Application to Intravascular OCT Image Segmentation
Source: IEEE Access. Author manuscript; Available in PMC 2021 Apr 6. (PMC8023588; doi:10.1109/access.2021.3058890)

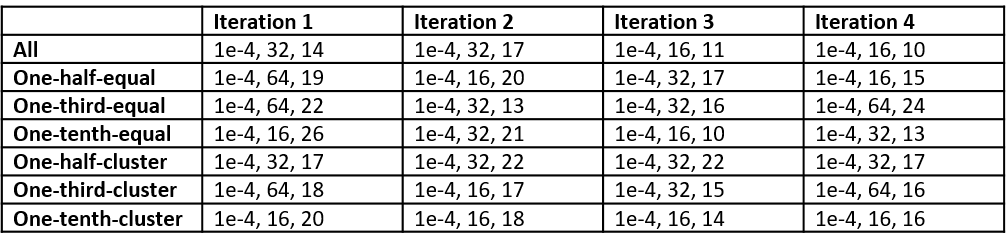

Supplement: access-3058890-mm [file NIHMS1681423-supplement-access-3058890-mm.zip › access-3058890-mm/Supplementary Material S2 Best hyper-parameter set (learning rate, batch size and number of epochs respectively) for all segmentation models created in this work.png]

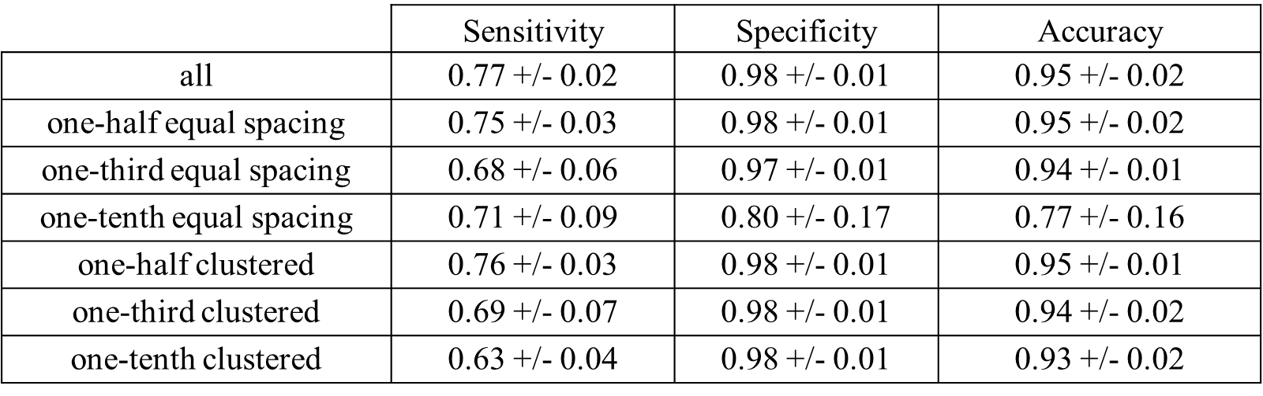

Supplement: access-3058890-mm [file NIHMS1681423-supplement-access-3058890-mm.zip › access-3058890-mm/Supplementary Material S3 Additional performance metrics of all models created in this work.png]

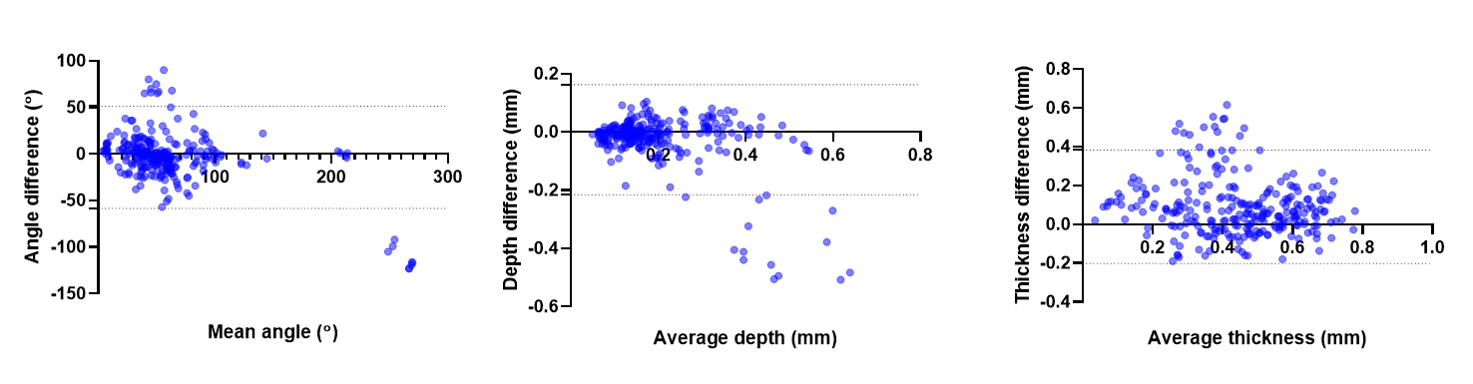

Supplement: access-3058890-mm [file NIHMS1681423-supplement-access-3058890-mm.zip › access-3058890-mm/Supplementary Material S4 Bland Altman analysis.png]

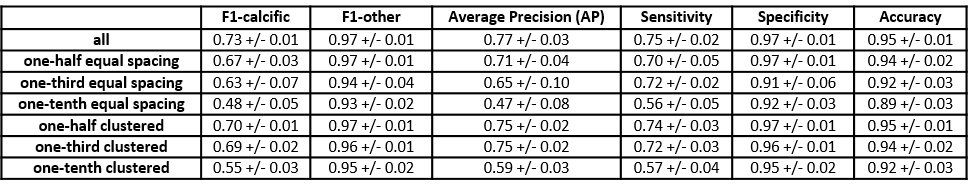

Supplement: access-3058890-mm [file NIHMS1681423-supplement-access-3058890-mm.zip › access-3058890-mm/Supplementary Material S7 Performance metrics of all models when trained with an alternate network architecture.png]
